# A population-based cohort study of sex and risk of severe outcomes in covid-19

**DOI:** 10.1007/s10654-022-00919-9

**Published:** 2022-10-27

**Authors:** Johanna Sieurin, Gunnar Brandén, Cecilia Magnusson, Maria-Pia Hergens, Kyriaki Kosidou

**Affiliations:** 1 Department of Global Public Health, Karolinska Institutet, Stockholm, Sweden; 2 Centre for Epidemiology and Community Medicine, Region Stockholm, Stockholm, Sweden; 3 Department of Communicable Disease Control and Prevention, Stockholm County Council, Stockholm, Sweden

**Keywords:** Sex, COVID-19, Morbidity, Mortality, Prospective cohort study

## Abstract

There is a male sex disadvantage in morbidity and mortality due to COVID-19. Proposed explanations to this disparity include gender-related health behaviors, differential distribution of comorbidities and biological sex differences. In this study, we investigated the association between sex and risk of severe COVID-19 while adjusting for comorbidities, socioeconomic factors, as well as unmeasured factors shared by cohabitants which are often left unadjusted. We conducted a total-population-based cohort study (n = 1,854,661) based on individual-level register data. Cox models was used to estimate the associations between sex and risk for severe COVID-19. We additionally used a within-household design and conditional Cox models aiming to account for unmeasured factors shared by cohabitants. A secondary aim was to compare the risk of COVID-19 related secondary outcomes between men and women hospitalized due to COVID-19 using logistic regression. Men were at higher risk for hospitalization (HR = 1.63;95%CI = 1.57–1.68), ICU admission (HR = 2.63;95%CI = 2.38–2.91) and death (HR = 1.81;95%CI = 1.68–1.95) due to COVID-19, based on fully adjusted models. However, the effect of sex varied significantly across age groups: Among people in their 50s, men had > four times higher risk of COVID-19 death. The within-household design did not provide any further explanation to the sex disparity. Among patients hospitalized due to COVID-19, men had an increased risk for viral pneumonia, acute respiratory distress syndrome, acute respiratory insufficiency, acute kidney injury, and sepsis which persisted in fully adjusted models. Recognition of the combined effect of sex and age on COVID-19 outcomes has implications for policy strategies to reduce the adverse effects of the disease.

**Supplementary Information:**

The online version contains supplementary material available at 10.1007/s10654-022-00919-9.

## Introduction

Since the outbreak of Coronavirus disease 2019 (COVID-19) caused by the virus SARS-CoV-2 detected in November 2019 and declared a pandemic by the World Health Organization in March 2020, a growing body of evidence reveals male sex as an important risk factor for severe COVID-19 outcomes. Globally, men are overrepresented among hospitalizations, intensive care unit (ICU) admissions and deaths due to COVID-19, despite similar infection rates between sexes.[1–4] However, the underlying causes for the observed sex disparity in severe COVID-19 outcomes remains unknown.

Apart from old age and male sex, comorbidities, including widespread cardiovascular and endocrine diseases, are known risk factors for severe COVID-19.[5] As the prevalence of many of these diseases differ between sexes, underlying comorbidities and related life-style factors have been suggested as a plausible explanation for the excess risk among men. Gender-based differences in attitudes and behaviors, such as COVID-19 risk perception, healthcare seeking behavior, and compliance to behaviors to avoid infection and spread of the disease,[6, 7] have also been suggested to contribute to the sex disparity. There are moreover biological sex differences with impact on the immune system that may explain the male vulnerability to severe COVID-19.[8, 9] Women generally elicit stronger innate and adaptive immune responses towards antigens,[8, 10–12] resulting in differences in prevalence and outcomes between men and women for several infectious, inflammatory, and autoimmune diseases; Generally, men are more vulnerable to infectious diseases, whereas women are more likely to develop autoimmune diseases. Studies from previous coronavirus outbreaks revealed that men are at higher risk for Severe Acute Respiratory Syndrome (SARS)[13] and Middle East Respiratory Syndrome (MERS).[14] There is also evidence that women in general develop a more robust humoral and cell-mediated immune response and report more adverse effects towards vaccination than men.[15–18] Similarly, differences between sexes in measured immune responses among COVID-19 patients have also been identified. Male sex has been associated with higher levels of proinflammatory cytokines and chemokines such as IL-6, IL-8, IL-18 and CCL5, higher neutrophil-lymphocyte ratios, lower absolute lymphocyte counts and a more robust induction of non-classical monocytes, whereas female sex has been associated with a more robust T-cell activation.[9, 19, 20] Thus, differences in immune responses have been suggested to explain the excess risk of severe COVID-19 outcomes among men, although the cause and effect between immune response and disease severity is not fully clear.

Numerous studies have indicated that male sex is a significant risk factor for severe COVID-19. Many studies are based on large, aggregated data with limited ability to adjust for important confounders, whereas other studies are smaller, hospital-based, and often suffer from selection bias and limited generalizability. Therefore, the main aim of this large population-based cohort study was to examine the sex disparity in the risk of hospitalization, ICU admission and death due to COVID-19 by utilizing the ability to link several health and administrative registers containing individual-level data on COVID-19 outcomes, as well as important comorbidities and socioeconomic factors. To analyze the sex disparity in severe COVID-19 outcomes further, we additionally used a within-household design in a subpopulation of the cohort aiming to adjust for unobserved factors shared by individuals living in the same household. This includes housing-related living conditions such as many social, economic and lifestyle related factors which are often shared by cohabitants to a large extent but are often left unadjusted despite being a potential source of omitted variable bias. The within-household design also partly accounts for SARS-CoV-2 exposure since much of the virus transmission occurs within households. A secondary aim of this study was to compare the risk of severe secondary outcomes related to COVID-19 between men and women hospitalized due to COVID-19.

## Methods

### Study design

We conducted a population-based prospective cohort study to examine the association between sex and risk for severe outcomes in COVID-19 defined as hospitalization, ICU admission or death due to COVID-19. The eligible study population was defined as all individuals aged 18 and older who were living in the region of greater Stockholm, Sweden, on March 1, 2020 (n = 1,854,666). The region of greater Stockholm represents more than one fifth of the entire population in Sweden. Five individuals with missing information on date of death were excluded resulting in a final study population of 1,854,661 individuals.

Through the unique personal identity number assigned to all Swedish citizens and residents intending to stay at least one year in Sweden,[21] we were able to perform unambiguous linkage between several population-based health and administrative registers for each individual in the cohort. Each individual was followed from March 1, 2020, until date of outcome event (i.e., hospitalization, ICU admission, or death due to COVID-19), emigration from the region of greater Stockholm, date of death, or to the end of the study period, whichever occurred first. The end of study period was defined as May 16, 2021 for hospitalization and ICU admission due to COVID-19 and December 31, 2020 for death due to COVID-19.

We further analyzed a cohabitation cohort to compare the risk of severe COVID-19 outcomes between men and women living within the same household. This comparison controlled for unknown and unmeasured factors shared among individuals living in the same household that might confound the association between sex and risk of severe COVID-19. Individuals living together evidently share the same housing-related living conditions, as well as many other social, economic and lifestyle related factors to a large extent. Further, since much of the transmission of SARS-CoV-2 occur within households, individuals living together have a similar risk of SARS-CoV-2 exposure. To create our cohabiting cohort, we started with the 1,854,661 individuals included in the population-based cohort and proceeded to exclude 4,173 individuals (< 1%) with missing information on household-id, and 564,842 individuals (30%) who were not living together with another adult person of the opposite sex. Among the 1,285,646 individuals that remained, we only included the oldest opposite-sexed pair of individuals living within the same household, with a maximum age difference of 5 years. In this step, 526,714 individuals (28%) were excluded, which resulted in a cohabiting cohort consisting of 758,932 individuals.

### Main outcomes

We considered three main COVID-19 related outcomes in this study: hospitalization, ICU admission and death due to COVID-19.

We derived information on hospitalizations due to COVID-19 from the Stockholm Regional Healthcare Data Warehouse (Vårdanalysdatabasen [VAL]) which provides information on all admissions to hospital as well as all healthcare visits in primary and secondary care (defined as specialist outpatient care).[22] Each record contains one primary diagnosis and up to nine additional diagnoses coded according to the International Classifications of Diseases (ICD), date of hospital admission and discharge or date of healthcare visit among others. We defined hospitalization due to COVID-19 as the first date of admission to hospital with COVID-19 (ICD-10 code U07.1 or U07.2) recorded as a primary diagnosis in the VAL-database.

Admissions to ICU were identified from the Swedish Intensive Care Registry (SIR), a national quality register for intensive care in Sweden including all ICUs in the region of greater Stockholm.[23, 24] We defined admission to ICU due to COVID-19 as a record in the SIR during a hospital episode with a COVID-19 diagnosis (ICD-10 code U07.1 or U07.2).

Data on deaths due to COVID-19 was retrieved from the cause of death register (CDR) held by the Swedish National Board of Health and Welfare. The CDR has nationwide coverage and contains information from death records including the underlying and contributory causes of death coded according to the ICD.[25] We defined death due to COVID-19 as a recorded diagnosis of COVID-19 (ICD-10 code U07.1 or U07.2) as the underlying or contributory cause of death in the CDR.

To assess the robustness of our results, we also used a more inclusive definition of COVID-19 related hospitalizations. This definition held that COVID-19 could be registered as either a primary or a secondary diagnosis. We also performed a similar robustness check regarding COVID-19 related deaths, using a more restrictive definition that held that COVID-19 had to be an underlying cause of death.

### Independent variables

Several comorbid conditions or history of diseases are associated with an increased risk for severe COVID-19 outcomes. Thus, we attained information on disease history, including possible comorbidities, for all study participants by searching recorded diagnoses in the VAL-database for the following diseases with ICD codes within parentheses; essential hypertension (I10.9), ischemic heart diseases (I20-25), heart failure (I50), stroke (I60-63), chronic obstructive pulmonary disease (COPD) (J44), asthma (J45), type 2 diabetes (E11), obesity (E66), chronic kidney disease (N18), chronic liver disease (K70), and cancer (C00-C97). We described those having an aforementioned disease recorded as a primary or secondary diagnosis on at least one consultation in primary or secondary care or during hospitalization from five years prior study entry until end of study period as having a comorbidity. For cases identified with COVID-19, only diagnoses recorded before the first COVID-19 diagnosis were considered.

We derived data on educational attainment and disposable household income from the Longitudinal integrated database for health insurance and labor market studies (LISA) maintained by Statistics Sweden.[26] Highest educational attainment was categorized into four groups: primary schooling (9 years or fewer), secondary education (9–12 years), higher education (> 12 years), and missing. Non-missing disposable family income was grouped into tertiles, while a separate group was made for those with missing information. The first tertile contained the lowest third of the income distribution, and the third tertile contained the highest third of the income distribution.

### Clinical outcomes of COVID-19

A second aim of this study was to compare outcomes in terms of diagnoses associated with severe illness due to COVID-19 between men and women hospitalized with COVID-19 as a main diagnosis. The following diagnoses were considered with ICD-10 codes within parentheses: viral pneumonia (J12.8-J12.9, J18.9), acute respiratory distress syndrome (ARDS) (J80.9), acute respiratory insufficiency (J96.0), acute kidney injury (N17), acute hepatic injury (K72.0, K72.9), acute cardiac injury (I21), sepsis (R65.1), septic shock (R57.2, R57.9), pulmonary embolism (I26), cerebral infarct (I63), embolism and thrombosis (I74, I80, I82). To be defined as a clinical outcome of COVID-19, the diagnosis had to be diagnosed during a hospital episode with COVID-19 as the main diagnosis. Cases with sequential admissions with a readmission within one day of the previous discharge were considered as one hospital episode to account for transfer of patients within and between hospitals.

### Statistical analysis

We used Cox proportional hazard regression models to estimate hazard ratios (HRs) with 95% confidence internals (CIs) for the risk of severe outcomes in COVID-19 associated with sex, using time after the start of follow-up in days as the underlying time scale. We adjusted all models for age using restricted cubic splines with 5 knots and then sequentially added other potential confounders including comorbidities and socioeconomic factors. We also tested for interactions between age and sex on COVID-19 outcomes and estimated separate HRs for men compared to women for different age groups (< 40, 40–49, 50–59, 60–69, 70–79 and 80 + years). The proportional hazards assumption was assessed graphically using log–log plots and tested based on Schoenfeld residuals. To further examine whether the effect of sex varied during follow-up, we split the follow-up time between the first and second wave of the pandemic (end of August, 2020) and included an interaction term between sex and time-period in our models.

We repeated the main analyses in the cohabitation cohort, using a standard Cox proportional hazards model, with partially and fully adjusted models as described above. To account for unobserved characteristics shared by men and women living in the same household, we performed corresponding analyses using conditional Cox models, stratified by household id. This approach allows each pair of cohabitants to have an individual baseline hazard function, while at the same time allowing it to vary between pairs. An attenuation of an estimate suggests that household-related factors contribute to the association, while the persistence of an estimate suggests that the association between sex and risk for severe COVID-19 is independent from factors shared within households.

We used multivariate logistic regression to explore if outcomes, in terms of diagnoses associated with severe COVID-19, differed between men and women hospitalized due to COVID-19. All tests were 2-sided and p < 0.05 was considered statistically significant. All analyses were conducted using Stata Statistical Software: Release 14 (StataCorp LP, College Station, Texas).

## Results

The study population consisted of 1,854,661 individuals, representing the whole adult population of Stockholm County, who were followed for hospitalizations and ICU admissions due to COVID-19 between March 1, 2020 and May 16, 2021 with an average follow-up time of 432 days. During 2,192,925 person-years under observation, 16,475 hospitalizations and 1,974 ICU admissions occurred. The study cohort was also followed for deaths due to COVID-19 between March 1, 2020 and December 31, 2021, with an average follow up time of 302 days amounting to a total of 1,531,833 person-years under observation. During this period 3,281 deaths due to COVID-19 occurred. Table 1 shows the distribution of population at risk, hospitalizations, ICU admissions and deaths due to COVID-19 for the variables used in our analyses. The incidence rate for hospitalization, ICU admission and death due to COVID-19 was higher in men, people with older age, comorbidities, low income, and low education level.

**Table 1:** Characteristics of study population, N % IR

	Observations			COVID-19		COVID-19		COVID-19	
	at baseline			Hospitalizations		ICU		Deaths	
	N	%		N	IR	N	IR	N	IR
**Total cohort**	1,854,661	100		16,475	7.5	1974	0.9	3281	2.1
*Men*	917,570	49.5		9,520	8.8	1396	1.3	1763	2.3
*Women*	937,091	50.5		6,955	6.3	578	0.5	1518	2.0
**Age groups**									
*18–39*	688,600	37.1		1,086	1.3	123	0.2	14	0.02
*40–49*	323,736	17.5		1,573	4.1	179	0.5	24	0.1
*50–59*	303,762	16.4		2,725	7.5	395	1.1	86	0.3
*60–69*	227,150	12.3		3,060	11.3	588	2.2	206	1.1
*70–79*	195,371	10.5		3,380	14.7	532	2.3	632	3.9
*80+*	116,042	6.3		4,651	35.8	157	1.2	2319	25.0
Comorbidities*									
*Essential hypertension*	332,039	17.9		8441	21.8	982	2.5	2275	8.3
*Ischemic heart diseases*	52,485	2.8		2213	37.1	205	3.4	757	17.8
*Heart failure*	34,837	1.9		2213	59.0	157	4.1	973	35.6
*Stroke*	21,339	1.2		931	39.4	59	2.5	427	25.2
*COPD*	35,529	1.9		1580	39.3	129	3.2	493	17.2
*Asthma*	94,658	5.1		1885	16.9	238	2.1	262	3.3
*Type 2 diabetes*	99,899	5.4		3794	32.9	513	4.4	912	11.1
*Obesity*	71,081	3.8		1784	21.3	309	3.7	219	3.7
*Chronic kidney disease*	31,043	1.7		1771	51.7	130	3.7	717	29.1
*Chronic liver disease*	11,077	0.6		319	25.2	56	4.4	68	7.6
*Cancer*	93,825	5.1		2330	21.6	211	1.9	729	9.6
**Income**									
*Income: 1st tertile*	600,962	32.4		7752	11.0	854	1.2	2017	4.1
*Income: 2nd tertile*	601,234	32.4		4839	6.8	601	0.8	827	1.7
*Income: 3rd tertile*	600,602	32.4		3706	5.2	503	0.7	421	0.8
*Missing*	51,863	2.8		178	3.0	16	0.3	16	0.4
**Education level**									
*Primary*	289,420	15.6		4048	11.9	468	1.4	1045	4.4
*Secondary*	664,928	35.9		6412	8.2	847	1.1	1319	2.4
*Post-secondary*	838,314	45.2		5394	5.4	597	0.6	761	1.1
*Missing*	61,999	3.3		621	8.7	62	0.9	156	3.1
**Work status**									
*At work < 50%*	481,310	26.0		1911	3.3	235	0.4	69	0.2
*At work > 50%*	518,280	27.9		3485	5.7	578	0.9	123	0.3
*At work > 50% health/social care*	119,266	6.4		917	6.5	127	0.9	27	0.3
*Missing < 65 years*	427,862	23.1		1995	4.0	312	0.6	98	0.3
*Missing > 65 years*	307,943	16.6		8167	22.9	722	2.0	2964	11.8

ICU = Intensive care unit, IR = Rate per 1000 person-years, COPD = Chronic obstructive pulmonary disease

* Number of individuals diagnosed with the listed diseases during the study-period or during the last five years prior the first date of the study. For COVID-19 cases, only records diagnosed before the first COVID-19 diagnosis are included

In Table 2, we present the results from multivariate Cox regressions of the risk for severe outcomes in COVID-19 among men compared to women. Male sex was associated with higher rates of hospitalization due to COVID-19, with HRs that remained stable around 1.6 in both partially and fully adjusted models (fully adjusted HR: 1.63; 95% CI: 1.57–1.68). However, there was a statistically significant (p < 0.05) interaction with age; men’s increased risk was most pronounced among middle aged people. Men aged 40–49 experienced a twice as large risk for hospitalization due to COVID-19 compared to women of the same age, while young adult men (18–39 years) only experienced 25% larger risk and men of old age (> 80 years) only experienced a 40% larger risk. The pattern was similar for ICU admissions due to COVID-19. Overall, men experienced approximately 2.6 times higher risk of ICU admission due to COVID-19, and the point estimate remained stable and statistically significant in all models (fully adjusted HR: 2.63; 95% CI: 2.38–2.91). There was again a statistically significant (p < 0.05) interaction with age. Young adult men (18–39 years) did not experience a statistically significant increased risk compared to women of the same age, whereas for all ages above 40, men experienced a three times larger risk for ICU admission. Finally, the pattern was also similar for death due to COVID-19. Overall, men experienced approximately 80% greater risk of death due to COVID-19 (fully adjusted HR: 1.81; 95% CI: 1.68–1.95). Again, this effect varied significantly (p < 0.05) across age groups with the most pronounced difference observed among men and women aged 50–59 years, where men experienced a ~ 4.6 times higher risk of death. The stark difference in risk of death then decreased with increasing age, settling at ~ 1.6 larger risk for men older than 80 years compared to women of the same age. We did not find any increased risk of death due to COVID-19 among men younger than 50 years of age, however, there were very few deaths in these age groups. Overall, adjusting for comorbidities and socioeconomic factors did not substantially change the point estimates for any of the outcomes; neither for the total cohort nor for any specific age group (Table 2).

**Table 2:** Hazard ratios of hospitalization, ICU admission, and death due to COVID-19 for men compared to women

COVID-19	Model 1				Model 2				Model 3		
outcomes	HR	95% CI			HR	95% CI			HR	95% CI	
Hospitalization											
Total cohort	1.62	1.57	1.67		1.54	1.49	1.59		1.63	1.57	1.68
By Age groups											
18–39	1.17	1.04	1.32		1.23	1.10	1.39		1.26	1.12	1.42
40–49	1.87	1.69	2.08		1.95	1.76	2.17		2.03	1.83	2.26
50–59	1.72	1.59	1.86		1.72	1.59	1.86		1.79	1.65	1.93
60–69	1.83	1.70	1.97		1.74	1.62	1.87		1.81	1.68	1.95
70–79	1.73	1.62	1.86		1.57	1.47	1.68		1.67	1.56	1.79
80+	1.43	1.35	1.52		1.30	1.22	1.38		1.41	1.33	1.50
ICU admission											
Total cohort	2.58	2.34	2.84		2.51	2.27	2.77		2.63	2.38	2.91
By Age groups											
18–39	0.96	0.67	1.36		1.03	0.73	1.47		1.05	0.73	1.49
40–49	2.40	1.74	3.31		2.53	1.83	3.50		2.62	1.90	3.63
50–59	2.98	2.37	3.74		2.99	2.38	3.76		3.11	2.47	3.91
60–69	3.07	2.54	3.70		2.94	2.44	3.55		3.07	2.54	3.72
70–79	2.46	2.05	2.95		2.28	1.90	2.75		2.45	2.03	2.94
80+	2.97	2.12	4.14		2.81	2.01	3.94		3.08	2.20	4.31
Deaths											
Total cohort	1.87	1.74	2.00		1.67	1.55	1.79		1.81	1.68	1.95
By Age groups											
18–39	0.73	0.25	2.11		0.76	0.26	2.19		0.77	0.27	2.22
40–49	0.98	0.44	2.18		1.00	0.45	2.23		1.01	0.45	2.25
50–59	4.71	2.70	8.22		4.65	2.66	8.11		4.61	2.64	8.06
60–69	3.09	2.25	4.24		2.91	2.12	3.99		2.92	2.12	4.02
70–79	2.15	1.82	2.53		1.93	1.64	2.28		2.07	1.75	2.44
80+	1.69	1.55	1.83		1.49	1.37	1.63		1.65	1.51	1.80

Abbreviations: HR = Hazard ratio, CI = Confidence intervals. ICU = intensive care unit.

Model 1: adjusted for age.

Model 2: adjusted for age and co-morbidities (hypertension, ischemic heart diseases, heart failure, stroke, COPD, asthma, type 2 diabetes, obesity, chronic kidney disease, chronic liver disease, cancer).

Model 3: adjusted for age, co-morbidities as in model 2 and education level, income, and work status.

Generally, we found no evidence that sex in models with hospitalizations and deaths related to covid-19 violated the proportional hazards assumption. However, in models with covid-19 related ICU admissions as the outcome, sex seemed to violate the proportional hazards assumption. This was addressed in sensitivity models including an interaction term between sex and time-period. Expectedly, there were no statistically significant interactions between sex and time-period (i.e., the first and second wave of the pandemic) for covid-19 related hospitalizations and deaths, and the HRs for sex were essentially the same during the two time-periods. For covid-19 related ICU admissions, the effect of male sex was slightly lower during the second wave (HR: 2.37; 95% CI: 2.08–2.71) compared to the first wave (HR: 2.98; 95% CI: 2.57–3.45). To assess the robustness of our results, we estimated all our models using a more inclusive definition of COVID-19 related hospitalizations. Reassuringly, our results using this definition of COVID-19 related hospitalization were similar with one exception; women at reproductive age (18–40 years) had an increased risk for hospitalization related to COVID-19 compared to men of the same age (S1 Table). However, this was solely explained by pregnant women receiving pregnancy and delivery-related health care as the main diagnosis and COVID-19 registered as a secondary diagnosis, which means that the difference in the results likely reflects intensive testing and reporting among this otherwise generally healthy female population.[27] We also performed a similar robustness check regarding COVID-19 related deaths, but with a more restrictive definition of deaths due to COVID-19. Again, the results were very similar using this more restrictive definition but again with one exception: men aged 50–59 had an even more pronounced risk of severe COVID-19 compared to women of the same age (S1 Table).

In Table 3 we present the results from multivariate and conditional Cox regressions based on the cohort of opposite-sexed pairs of similar age living within the same household (n = 758,932 individuals). The magnitudes of men’s increased risk for hospitalization, ICU admission and death due to COVID-19 are the same among people included in the total cohort and the cohort of cohabiting persons; the estimates from standard Cox regressions based on the overall cohort (Table 2, Model 1 and 3) did not differ substantially from the corresponding analyses for the cohabiting cohort (Table 3, Model 1 and 2). Furthermore, adjusting for unobserved factors shared within households (Table 3, Model 3) and further adjustment for comorbidities and socioeconomic factors (Table 3, Model 4) in conditional Cox models did not have a substantial effect on the point estimates either.

**Table 3:** Sub-analysis of cohabiting-cohort

	Cox regression								Conditional Cox regression						
COVID-19	Model 1^a^				Model 2^b^				Model 3^c^				Model 4^d^		
Outcome	HR	95% CI			HR	95% CI			HR	95% CI			HR	95% CI	
Hospitalization	1.58	1.50	1.67		1.55	1.47	1.65		1.67	1.57	1.78		1.66	1.55	1.78
ICU admission	2.45	2.08	2.89		2.37	2.00	2.81		2.79	2.28	3.41		2.85	2.24	3.62
Death	2.11	1.78	2.49		1.85	1.56	2.20		2.31	1.89	2.82		2.13	1.68	2.69

Abbreviations: HR = Hazard ratio, CI = Confidence intervals, ICU = intensive care unit

^a^ Adjusted for age

^b^ Adjusted for age, medical comorbidities, education level, income and work status defined by potential virus exposure

^c^ Adjusted for age and household-id through design

^d^ As model 3 and additionally adjusted for medical comorbidities, education level, income and work status

In Table 4 we present the results from logistic regression models examining the association between sex and diagnosis of acute and severe secondary outcomes among hospitalized COVID-19 patients. After adjusting for age, men had higher odds of being diagnosed with viral pneumonia, acute respiratory distress syndrome, acute respiratory insufficiency, acute kidney injury, acute cardiac injury, sepsis, and cerebral infarcts. After adjusting for comorbidities and socioeconomic factors, the association of sex with acute cardiac injury and cerebral infarcts became insignificant as the point estimates dropped closer to one. In contrast, the association of sex with viral pneumonia, acute respiratory distress syndrome, acute respiratory insufficiency, acute kidney injury, and sepsis all remained significant, and the magnitude of the estimates remained essentially unchanged.

**Table 4:** Multivariable logistic regression analysis of the association between sex and COVID-19 related complications among hospitalized COVID-19 patients

	Women			Men				
	n	%		n	%		OR^a^ (95% CI)	OR^b^ (95% CI)
Total No. patients	6,955			9,520				
Pneumonia	2,418	34.8		3,944	41.4		1.25 (1.17–1.33)	1.28 (1.20–1.37)
ARDS	514	7.4		1,149	12.1		1.54 (1.38–1.72)	1.62 (1.46–1.84)
ARI	1,105	15.9		1,636	17.2		1.22 (1.12–1.33)	1.27 (1.17–1.38)
Acute kidney injury	287	4.1		607	6.4		1.72 (1.48–1.99)	1.70 (1.46–1.98)
Acute hepatic injury	18	0.3		21	0.2		0.77 (0.41–1.45)	0.95 (0.47–1.90)
Acute cardiac injury	55	0.8		99	1.0		1.64 (1.17–2.29)	1.37 (0.96–1.98)
Sepsis	166	2.4		323	3.4		1.44 (1.19–1.75)	1.42 (1.16–1.74)
Septic shock	45	0.7		101	1.1		1.39 (0.98-1-98)	1.42 (0.99–2.07)
Pulmonary embolism	291	4.2		446	4.7		1.06 (0.91–1.23)	1.07 (0.91–1.25)
Cerebral infarct	65	0.9		119	1.3		1.56 (1.14–2.11)	1.24 (0.90–1.73)
Embolism/thrombosis	69	1.0		110	1.2		1.10 (0.81–1.50)	1.14 (0.83–1.58)

Abbreviations: OR = Odds ratio, CI = Confidence intervals, ARDS = acute respiratory distress syndrome, ARI = acute respiratory insufficiency

^a^ Adjusted for age

^b^ Adjusted for age, comorbidities, education level, income, and work status

## Discussion

In this large population-based study, we have observed a substantial male sex disadvantage in severe morbidity and mortality due to COVID-19. In general, men’s excess risk was most pronounced among middle aged people (50–59 years), in particular for the risk of COVID-19 related death. Adjusting for several important comorbidities and socioeconomic factors did not attenuate the observed associations. Further, adjusting for unobserved factors shared by individuals in the same household in a conditional Cox analysis did not provide any further explanation to the sex disparity in severe COVID-19 outcomes. In addition, among patients hospitalized due to COVID-19, men had an increased risk for complications associated with COVID-19 such as viral pneumonia, acute respiratory distress syndrome, acute respiratory insufficiency, acute kidney injury, and sepsis.

Our finding that men are at higher risk of severe COVID-19 infection and death compared to women, and that the increased relative risk varies across age-groups is consistent with previous studies.[1, 3, 19] The age-sex pattern of a pronounced higher relative risk for men around middle age has been hypothesized to be (at least partially) caused by the differential distribution of comorbidities across sexes and age strata. Women are generally reported to have a higher comorbidity burden, especially at older ages. However, women tend to suffer from more non-fatal chronic conditions such as migraine, depression, autoimmune and musculoskeletal diseases, whereas men have more life-threatening conditions such as cardiovascular diseases, hypertension, chronic lung diseases and type-2 diabetes,[28, 29] which are associated with a worse progression of COVID-19.[5] Thus, a higher burden of specific comorbidities in men may explain the markedly increased risk for severe COVID-19 outcomes around middle age, whereas a reduction of the increased relative risk for men at older ages may be explained by a survival effect leaving only the healthiest men to survive to old age. However, adjustments for comorbidities and socioeconomic factors did not attenuate the association between sex and severe COVID-19 outcomes in any age group. This finding is in line with previous studies on case fatality among patients with a confirmed infection reporting an increased risk among men independent of comorbidities, demographics, and health behaviors.[19, 30].

Gender-related differences in occupations, health-behaviors and attitudes have also been suggested to partly explain men’s increased risk for COVID-19 outcomes.[1] Studies have shown that women are more likely to perceive COVID-19 as a serious health problem and to agree and comply with restraining public policies.[6] However, such behaviors are primarily associated with risk for infection, whereas results from large meta-analyses and epidemiological surveillance demonstrate that COVID-19 infection rates are similar between sexes.[2, 4] Further, the test positivity rate among asymptomatic individuals has also been shown to be similar between sexes,[19] or even higher in women.[31] Therefore, the increased risk for morbidity and mortality in COVID-19 among men does not seem to be explained by higher risk of SARS-CoV-2 exposure or susceptibility. Our finding that among hospitalized patients with COVID-19, men had an increased risk for COVID-19 related complications (i.e., viral pneumonia, acute respiratory distress syndrome, acute respiratory insufficiency, acute kidney injury, and sepsis) independent of previous comorbidities and socioeconomic factors, also suggests that men have a higher risk of severe disease progression, rather than just increased virus exposure or susceptibility. It has also been suggested that gender-related social norms may cause men to postpone seeking out medical treatment, thereby experiencing more severe COVID-19 due to this delay. However, previous studies have found indications both for and against this tendency.[19, 32] Since we have not been able to adjust for these types of behaviors, we cannot rule out that delayed treatment among men partially explains our results. However, data from countries worldwide show that the sex disparity in COVID-19 case fatality and intensive therapy unit admissions is a global phenomenon with relatively homogenous relative risk estimates,[1, 4, 10] whereas gender differences in social behavior, life-style factors and comorbidities vary across countries. In addition, results from the within-household design provided no further explanation to the sex disparity, despite accounting for housing-related living conditions, including many social and lifestyle related factors to a large extent, as well as exposure to SARS-CoV-2, given that much of the transmission of SARS-CoV-2 occur within households.[33] Thus, the within-household design presumably partly accounts for gender-related differences, especially those who are related to risk of infection, even though gender-related differences in attitudes and behavior exist within households. Thus, we find it unlikely that gender differences in social behaviors is a driver of men’s increased risk in severe COVID-19 outcomes.

The observed sex disparity in COVID-19 outcomes could be a consequence of several biological sex differences, such as sex steroid hormone levels, sex chromosomes, differential genetic expression, and differences in the immune function, whereof some may interact with age.[10] Sex steroid hormones have known immunomodulatory functions; whereas estrogens may have both pro- and anti-inflammatory effects, androgens mainly act immunosuppressive.[34–36] Experimental animal studies support a beneficial immunomodulatory effect of estrogens towards coronaviruses,[37, 38] and epidemiological evidence indicates that hormone replacement treatment with estradiol may reduce COVID-19 mortality in post-menopausal women.[39] Although less studied, there are also some epidemiological indications of a potential negative effect of androgens on risk of COVID-19 infection, as a positive effect of anti-androgen treatment among male prostate cancer patients was observed.[40].

Women may also benefit from having two X-chromosomes that encode a large number of immune-related genes.[41, 42] As carriers of a single X-chromosome, men are more vulnerable for X-linked mutations, compared to women who are mosaic for X-linked genes. Due to random X-inactivation in females, there is usually no difference in dosage of X-linked gene products between sexes. However, incomplete inactivation of X-linked genes may occur at varying degree between genes and individuals,[43] and have been associated with higher prevalence of autoimmune diseases in women. For example, enhanced Toll-like receptor 7 (TLR7) gene expression has been associated with increased risk for SLE and other autoimmune disorders in women.[44] TLR7 is a pattern recognition receptor used to detect single stranded RNA, including coronaviruses. Ligand binding to TLR7 results in an increased production of type I interferons, which is an important mechanism of the innate immune system towards virus infections found to be stronger in women.[45, 46] In case studies, supposed loss-of-function mutations in *TLR7* have been identified in young men with severe or fatal outcomes in COVID-19, without any known predisposing risk factors.[47, 48] A subsequent study screening for X-linked mutations in men, identified deleterious *TLR7* variants in men with unexplained severe COVID-19, whereas none were identified among the mildly or asymptomatically infected men.[49] Angiotensin-converting enzyme 2 (ACE2) is the entry receptor for SARS-CoV-2 and the *ACE2* gene is also coded on the X-chromosome and has been found to be regulated by estrogens.[50]ACE2 has a crucial role in the renin-angiotensin-aldosterone system (RAAS) which regulate blood pressure and electrolyte homeostasis. Thus, it has also been speculated that sex-derived differences in *ACE2* expression and regulation may in part explain differences in COVID-19 outcomes.[51, 52].

Severe COVID-19 is associated with a state of dysregulated immune responses associated with excessive production of inflammatory cytokines and chemokines, inflammatory cell infiltration in the lungs, a so-called cytokine storm, causing lung injury and respiratory distress, together with lymphopenia. Deaths due to COVID-19 usually result from acute respiratory distress syndrome, acute respiratory failure, coagulopathy, organ failure and septic shock.[53] We found that men hospitalized due to COVID-19 were at higher risk of acute cardiac injury and cerebral infarcts compared to women, but this effect seemed to be confounded by underlying comorbidities. In contrast, we also found that male COVID-19 patients had a higher risk of developing pneumonia, acute respiratory distress syndrome, acute respiratory insufficiency, acute kidney injury, and sepsis compared to female patients, which seemed to be independent of underlying comorbidities and socioeconomic factors.

A major strength of this study is the total population-based setting with complete coverage of COVID-19 related hospitalizations and deaths, why this study is not subjected to selection bias. Another important strength is the use of administrative register data on socioeconomic factors. For example, these included information on housing arrangements that enabled the novel use of within-household comparisons to address unknown and unmeasured confounders shared by cohabitants in the study of sex disparities in severe COVID-19 outcomes. Further, the unique Stockholm Regional Healthcare Data Warehouse, which contains information on hospitalizations as well as data from primary and secondary care, improved our capability to identify individuals with important comorbidities, compared to using only hospital-based data.

There are also limitations to consider. This is an observational study, and as such we cannot determine the causal relationships that underlies the male sex disadvantage in morbidity and mortality due to COVID-19. This study could have been subjected to surveillance bias as men generally receive more specialist inpatient care, which could increase the chance of being detected with COVID-19 in health registers. To minimize this issue, we defined COVID-19 hospitalizations based on main diagnosis only. The accuracy of COVID-19 diagnoses in Swedish health and cause of death registers is presumably high but have not been formally validated. Sensitivity of hospital diagnoses as a measure of severe COVID-19 morbidity among older patients may be an issue since age discrimination in access to care has occurred.[54] Although it has not been shown that this age discrimination differs by sex, results of hospitalizations and ICU admissions among elderly should be interpreted more carefully. Some cases of comorbidity may have been misclassified. However, this is likely nondifferential with regard to sex. On the other hand, under-diagnosis, especially of less severe comorbidities, may differ between sexes and introduce bias. Residual confounding by life-style factors and comorbidities, especially mild cases not identified by registers, may be a limitation. However, these factors are likely captured by adjustments for socioeconomic factors and factors shared within households to some extent, though adjustments for these factors did not notably change the observed estimates. Emerging evidence seems to support an association between smoking and more severe COVID-19 outcomes.[55, 56] However, more women than men smoke daily in Sweden, although the difference is marginal.[57] Therefore, it is not likely that smoking status is a major driver of the observed sex disparity. Another consideration is that the follow-up period for hospitalizations and ICU admissions (ending on May 16, 2021) overlaps with the introduction of vaccination against SARS-CoV-2, which started in week 52, 2020. The vaccination coverage increased slowly during the first quarter of 2021 and thereafter started to increase more steeply. At the end of our study period, almost 10% had received two doses and about 30% had received at least one dose. Thus, vaccination status could potentially affect our results for hospitalizations and ICU admissions. However, results from our sensitivity analysis showed that HRs were the same during the first (before vaccination was initiated) and second wave (when the vaccination started) of the pandemic. Thus, it is unlikely that vaccination status interferes with our results. It is also unlikely that other factors that vary over time or between pandemic waves, such as stochastic transmission of the virus to different groups of people and new mutations and strain variants emerging, affect the sex ratio observed in our results.

Our results confirm that men have an excess risk of hospitalization, ICU admission and death due to COVID-19. This sex disparity varies across age groups and is most pronounced among middle aged men compared to women of the same age. Further, among hospitalized COVID-19 patients, men are more likely to be diagnosed with COVID-19 related complications. Different explanations have been suggested to explain men’s vulnerability to COVID-19, including gender related health behaviors, differential distribution of comorbidities and biological sex differences such as sex steroid hormones, genetic constitution, or differential gene expression. The reason for men’s excessive risk is likely multifactorial with a complex interplay between several factors. In this study we adjusted for multiple confounders, including important comorbidities, socioeconomic factors as well as unmeasured household-related factors. These adjustments had no substantial impact on men’s excess risk of severe COVID-19 outcomes. Thus, our results motivate a focus in future studies on biological mechanisms that influence the immune response towards SARS-CoV-2 to help explain women’s advantage in COVID-19. This study also highlights the importance to consider the combined effect of age and sex when defining those at highest risk of severe COVID-19 outcomes.

## Electronic supplementary material

Below is the link to the electronic supplementary material.

Supplementary Material 1

Supplementary Material 2
