# Supplementary material for: A population-based cohort study of sex and risk of severe outcomes in covid-19
Source: Eur J Epidemiol. 2022 Oct 27;37(11):1159–69. doi: 10.1007/s10654-022-00919-9 (PMC9607822; doi:10.1007/s10654-022-00919-9)
Supplement: Supplementary file 1 — Supplementary Material 1 [file 10654_2022_919_MOESM1_ESM.pdf]

Supplementary information

S1 Table. Hazard ratios of hospitalization due to COVID-19 (defined by COVID-19 as main or secondary diagnosis), and death due to COVID-19 (defined by COVID-19 as underlying cause of death) for men compared to women

COVID-19	Model 1			Model 2			Model 3		
**outcomes**	**HR**	**95% CI**		**HR**	**95% CI**		**HR**	**95% CI**	
**Hospitalization**									
Total cohort	1.51	1.47	1.55	1.42	1.38	1.46	1.50	1.45	1.54
By Age groups									
18-39	0.74	0.67	0.81	0.77	0.79	0.85	0.79	0.72	0.87
40-49	1.66	1.51	1.82	1.72	1.56	1.89	1.78	1.62	1.96
50-59	1.69	1.57	1.82	1.69	1.57	1.82	1.75	1.62	1.89
60-69	1.85	1.73	1.99	1.75	1.63	1.87	1.81	1.69	1.95
70-79	1.69	1.59	1.80	1.51	1.42	1.61	1.60	1.50	1.71
80+	1.43	1.35	1.51	1.26	1.20	1.33	1.37	1.30	1.45
**Deaths**									
Total cohort	1.93	1.79	2.07	1.74	1.62	1.88	1.89	1.75	2.04
By Age groups									
18-39	0.56	0.16	1.91	0.58	0.17	1.99	0.59	0.17	2.02
40-49	2.35	0.83	6.67	2.41	0.85	6.84	2.42	0.85	6.87
50-59	8.21	3.94	17.1	8.12	3.90	16.92	8.03	3.85	16.7
60-69	3.41	2.42	4.79	3.22	2.29	4.53	3.22	2.28	4.54
70-79	2.16	1.81	2.57	1.96	1.65	2.34	2.10	1.76	2.50
80+	1.72	1.58	1.88	1.55	1.41	1.69	1.71	1.56	1.87

Abbreviations: HR = Hazard ratio, CI = Confidence intervals, ICU = intensive care unit

Model 1: adjusted for age

Model 2: adjusted for age and co-morbidities (hypertension, ischemic heart diseases, heart failure, stroke, COPD, asthma, type 2 diabetes, obesity, chronic kidney disease, chronic liver disease, cancer)

Model 3: adjusted for age, co-morbidities as in model 2 and education level, income, and work status.
