# Supplementary material for: A population-based cohort study of sex and risk of severe outcomes in covid-19
Source: Eur J Epidemiol. 2022 Oct 27;37(11):1159–69. doi: 10.1007/s10654-022-00919-9 (PMC9607822; doi:10.1007/s10654-022-00919-9)
Supplement: Supplementary file 2 — Supplementary Material 2 [file 10654_2022_919_MOESM2_ESM.docx]

# Supplementary information

### **S1 Table. Hazard ratios of hospitalization due to COVID-19 (defined by COVID-19 as main or secondary diagnosis), and death due to COVID-19 (defined by COVID-19 as underlying cause of death) for men compared to women**

| **COVID-19** | **Model 1** | |  |  | **Model 2** | |  |  | **Model 3** | |  |
| --- | --- | --- | --- | --- | --- | --- | --- | --- | --- | --- | --- |
| **outcomes** | **HR** | **95% CI** | |  | **HR** | **95% CI** | |  | **HR** | **95% CI** | |
| **Hospitalization** |  |  |  |  |  |  |  |  |  |  |  |
| *Total cohort* | 1.51 | 1.47 | 1.55 |  | 1.42 | 1.38 | 1.46 |  | 1.50 | 1.45 | 1.54 |
| By Age groups |  |  |  |  |  |  |  |  |  |  |  |
| *18-39* | 0.74 | 0.67 | 0.81 |  | 0.77 | 0.79 | 0.85 |  | 0.79 | 0.72 | 0.87 |
| *40-49* | 1.66 | 1.51 | 1.82 |  | 1.72 | 1.56 | 1.89 |  | 1.78 | 1.62 | 1.96 |
| *50-59* | 1.69 | 1.57 | 1.82 |  | 1.69 | 1.57 | 1.82 |  | 1.75 | 1.62 | 1.89 |
| *60-69* | 1.85 | 1.73 | 1.99 |  | 1.75 | 1.63 | 1.87 |  | 1.81 | 1.69 | 1.95 |
| *70-79* | 1.69 | 1.59 | 1.80 |  | 1.51 | 1.42 | 1.61 |  | 1.60 | 1.50 | 1.71 |
| *80+* | 1.43 | 1.35 | 1.51 |  | 1.26 | 1.20 | 1.33 |  | 1.37 | 1.30 | 1.45 |
| **Deaths** |  |  |  |  |  |  |  |  |  |  |  |
| *Total cohort* | 1.93 | 1.79 | 2.07 |  | 1.74 | 1.62 | 1.88 |  | 1.89 | 1.75 | 2.04 |
| By Age groups |  |  |  |  |  |  |  |  |  |  |  |
| *18-39* | 0.56 | 0.16 | 1.91 |  | 0.58 | 0.17 | 1.99 |  | 0.59 | 0.17 | 2.02 |
| *40-49* | 2.35 | 0.83 | 6.67 |  | 2.41 | 0.85 | 6.84 |  | 2.42 | 0.85 | 6.87 |
| *50-59* | 8.21 | 3.94 | 17.1 |  | 8.12 | 3.90 | 16.92 |  | 8.03 | 3.85 | 16.7 |
| *60-69* | 3.41 | 2.42 | 4.79 |  | 3.22 | 2.29 | 4.53 |  | 3.22 | 2.28 | 4.54 |
| *70-79* | 2.16 | 1.81 | 2.57 |  | 1.96 | 1.65 | 2.34 |  | 2.10 | 1.76 | 2.50 |
| *80+* | 1.72 | 1.58 | 1.88 |  | 1.55 | 1.41 | 1.69 |  | 1.71 | 1.56 | 1.87 |

Abbreviations: HR = Hazard ratio, CI = Confidence intervals, ICU = intensive care unit

Model 1: adjusted for age

Model 2: adjusted for age and co-morbidities (hypertension, ischemic heart diseases, heart failure, stroke, COPD, asthma, type 2 diabetes, obesity, chronic kidney disease, chronic liver disease, cancer)

Model 3: adjusted for age, co-morbidities as in model 2 and education level, income, and work status.
